# Immature adipocyte‐derived exosomes inhibit expression of muscle differentiation markers

**DOI:** 10.1002/2211-5463.13100

**Published:** 2021-02-16

**Authors:** Koichi Ojima, Susumu Muroya, Hiromu Wada, Kotaro Ogawa, Mika Oe, Koichi Takimoto, Takanori Nishimura

**Affiliations:** ^1^ Muscle Biology Research Unit Division of Animal Products Research National Institute of Livestock and Grassland Science NARO Tsukuba Japan; ^2^ Ion Channel Laboratory Department of Bioengineering Nagaoka University of Technology Nagaoka Japan; ^3^ Laboratory of Cell and Tissue Biology Research Faculty of Agriculture Graduate School of Agriculture, Hokkaido University Sapporo Japan

**Keywords:** adipocyte, exosome, miRNA, muscular dystrophy, skeletal muscle

## Abstract

Exosomes are released from a variety of cells to communicate with recipient cells. Exosomes contain microRNAs (miRNAs), which are noncoding RNAs that suppress target genes. Our previous proteomic study (*FEBS Open Bio* 2016, **6**, 816–826) demonstrated that 3T3‐L1 adipocytes secrete exosome components as well as growth factors, inspiring us to investigate what type of miRNA is involved in adipocyte‐secreted exosomes and what functions they carry out in recipient cells. Here, we profiled miRNAs in 3T3‐L1 adipocyte‐secreted exosomes and revealed suppression of muscle differentiation by adipocyte‐derived exosomes. Through our microarray analysis, we detected over 300 exosomal miRNAs during adipocyte differentiation. Exosomal miRNAs present during adipocyte differentiation included not only pro‐adipogenic miRNAs but also miRNAs associated with muscular dystrophy. Gene ontology analysis predicted that the target genes of miRNAs are associated primarily with transcriptional regulation. To further investigate whether adipocyte‐secreted exosomes regulate the expression levels of genes involved in muscle differentiation, we treated cultured myoblasts with adipocyte‐derived exosome fractions. Intriguingly, the expression levels of myogenic regulatory factors, Myog and Myf6, and other muscle differentiation markers, myosin heavy‐chain 3 and insulin‐like growth factor 2, were significantly downregulated in myoblasts treated with adipocyte‐derived exosomes. Immature adipocyte‐derived exosomes exhibited a stronger suppressive effect than mature adipocyte‐derived exosomes. Our results suggest that adipocytes suppress the expression levels of muscle differentiation‐associated genes in myoblasts via adipocyte‐secreted exosomes containing miRNAs.

## Graphical abstract

Exosomes contain miRNAs and function as a cell‐to‐cell communication tool. In our microarray analysis, adipocyte‐derived exosomes included not only pro‐adipogenic but also antimyogenic miRNAs. Myogenic differentiation markers were downregulated in myoblasts treated with adipocyte‐derived exosomes. In particular, immature adipocyte‐derived exosomes had a stronger suppressive effect on myoblasts. Our findings suggest that adipocytes modulate myogenic differentiation via exosomal miRNAs.

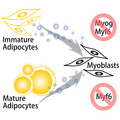

**Abbreviations**
D: differentiation day
GO: gene ontology
miRNA: microRNA

One of the main tissues that play a key role in the endocrine organs is adipose tissue. Adipocytes actively release a variety of factors including cytokines and growth factors, for example, leptin and adiponectin, which are referred to as adipokines [1]. Adipokines regulate the metabolic process in fat and other tissues since they have both proinflammatory and antiinflammatory activities. Like classical growth factors, most adipokines bind to their receptors on target cells to activate the intracellular signal transduction pathway in the target cells. Thus, adipocytes communicate with other types of cells as well as with other adipocytes through adipokine secretion and uptake.

Recently, exosomes have received considerable attention as cell–cell communication tools. Many types of cells release exosomes, which are tiny vesicles 30–200 nm in diameter that are found in blood, urine, and other body fluids [2, 3]. Importantly, exosomes contain proteins, lipids, mRNA, and microRNA (miRNA), which are generated in host cells [2]. miRNA is a single short RNA comprised of 21–25 nucleotides. Although miRNAs are coded in genes, they do not translate into proteins. Thus, miRNA is categorized as noncoding RNA. Functionally, miRNA binds to the 3′UTR region of its target mRNAs to destabilize and interfere with the translation of target mRNAs, leading to a reduction in the translational level of the target gene [4]. Transcriptional regulation via miRNA is found in a variety of biological activities such as cell proliferation, cell development, apoptosis, and metabolism [5, 6]. When released exosomes are incorporated into recipient cells, exosomes containing miRNA modulate the expression of their target genes in recipient cells [7, 8]. Therefore, in addition to the classical growth factors, exosomes are another important factor serving as a communication tool between cells.

Adipocytes are frequently deposited as visceral and subcutaneous adipose tissues, while ectopic adipocyte accumulation is found in the skeletal muscle tissues of muscular dystrophy patients [9, 10]. Muscular dystrophies are heterogeneous genetic diseases that cause progressive muscle weakness. The typical pathological muscle condition of muscular dystrophy includes progressive muscle degeneration, malfunction of muscle regeneration, progressive fibrosis, and the accumulation of adipocytes. In addition to disease, high intramuscular fat deposition is also observed in marbling cattle [11]. In both cases, adipocytes expand their cellular volume and increase their cell numbers between myofibers and the number of myofibers subsequently decreases. In the process of forming dystrophic muscle or marbling muscle, there must be intercellular communication between adipocytes and muscle cells. However, the mechanism by which two different types of cells communicate with each other is not yet fully understood.

In our search for factors that may function as cell‐to‐cell communication tools, we previously carried out a comprehensive analysis of secreted factors from 3T3‐L1 adipocytes [12]. This previous study revealed that differentiating 3T3‐L1 adipocytes release approximately 200 types of secreted peptides/proteins that contain extracellular matrix components, adipokines, etc. In addition to these peptides/proteins, we also found that typical exosomal component proteins were included in the list of 3T3‐L1‐secreted factors, which account for approximately 16% of all identified proteins. Furthermore, studies by other groups demonstrate that miRNAs are implicated in adipocyte differentiation [13, 14] and that exosomes are ideal vehicles for carrying miRNAs to modulate the translation of target genes in recipient cells [2]. These studies inspired us to hypothesize that adipocytes take advantage of exosomes to communicate and/or regulate their neighboring cells. In the present study, we carried out a comprehensive expression analysis of miRNA in differentiating adipocyte‐derived exosomes and evaluated the effect of that miRNA on cultured skeletal muscle cells. Our results demonstrate that cultured adipocytes actively release exosomes containing miRNAs to suppress or decelerate muscle cell differentiation.

## Materials and methods

### Exosome‐depleted sera

FBS (Biowest, Nuaillé, France) and horse serum (HS; Life Technologies Japan Ltd., Tokyo, Japan) were centrifuged at 15 000 ***g*** for 30 min to remove debris and further centrifuged at 110 000 ***g*** for 16 h to exclude intrinsic exosomes. Each serum was sterilized by passing it through 0.2‐μm filters (Sartorius, Göttingen, Germany). In this study, we used exosome‐depleted FBS and HS for cell culturing.

### Cell culturing

The 3T3‐L1 mouse adipocyte cell line was cultured as previously described in the literature with slight modification [12]. In brief, proliferating cells were kept on 90‐mm plastic dishes (Sumitomo Bakelite Co., Ltd., Tokyo, Japan) in growth medium [GM; 10% FBS in low‐glucose Dulbecco's modified Eagle's medium (DMEM); Life Technologies] for 3 days. To induce differentiation, the cells were cultured in adipocyte differentiation medium (DM)‐1 [10% FBS, 0.5 mm of 1‐methyl‐3‐isobutylxanthine (Nacalai Tesque Inc., Kyoto, Japan), 6.4 nm dexamethasone (Life Technologies), and 10 μg·mL^−1^ insulin (Wako Pure Chemical Industries, Ltd., Osaka, Japan) in high‐glucose DMEM]. After 2 days of culture, cells were switched to adipocyte DM‐2 (10% FBS in high‐glucose DMEM containing 10 μg·mL^−1^ insulin) for further culturing. All media were supplemented with 100 U·mL^−1^ penicillin and 0.1 mg·mL^−1^ streptomycin. Media were changed every other day. We defined Day *x* (D*x*) as day(s) after shifting cells to adipocyte DM‐1 from GM.

To measure DNA concentrations in cultured 3T3‐L1 cells, cell lysates were treated with proteinase K solution (Nacalai Tesque) and DNA concentration was measured with a spectrometer (Eppendorf BioSpectrometer, Eppendorf Japan, Tokyo, Japan).

Experimental animals were cared for as outlined in the guidelines of NARO. This study was approved by the committees. Mouse skeletal muscle culturing was described in our previous studies [15, 16]. In short, primary skeletal muscle cells were isolated from hindlimb muscles of C57Bl6N mouse. Connective tissue was removed carefully. Muscles were minced and digested with 0.2% collagenase type II (Worthington, Lakewood, NJ, USA) in Hanks' balanced salt solution for 30 min at 37 °C. Muscle suspension was passed through a 20‐G needle 10 times and further digested with 0.2% collagenase type II for 10 min. Subsequently, muscle suspension was filtered through a 100‐μm and a 40‐μm strainer. The cells were collected by centrifugation and plated on a noncoated dish for 1 h. Nonadherent cells were collected and plated on collagen‐coated dishes. The cells were cultured in proliferating medium [20% FBS in F10 with 2.5 ng·mL^−1^ human bFGF recombinant protein (Thermo Fisher Scientific, Tokyo, Japan)]. After some passages, these muscle cells were used for experiments. For exosome fraction treatment assay, muscle cells were cultured on Labtech chamber slides (Nalge Nunc International Inc., Rochester, NY, USA) or on 12‐well dishes (Sumitomo Bakelite) which were coated with Matrigel (Japan BD Bioscience, Tokyo, Japan). Cultured cells were shifted from muscle GM (10% FBS in DMEM) to muscle DM (5% HS in DMEM) to induce muscle differentiation. All media were supplemented with 100 U·mL^−1^ penicillin, 0.1 mg·mL^−1^ streptomycin, and 2 mm l‐glutamine (Life Technologies). Our previous studies demonstrate that isolated muscle cells showed normal myogenic properties [16, 17].

### Isolation of exosome fractions from 3T3‐L1 cell‐cultured media

Cultured media of 3T3‐L1 cells were collected on D0, 2, 4, 6, 8, 10, 12, 14, and 16 after switching to adipocyte DM‐1. At each stage of culture, cultured media were collected from 10–12 dishes with 90 mm in diameter, which amounts to 60–72 mL. A total of three independent experiments were carried out. Exosome fraction was isolated from cultured media following the ultracentrifugation method described in our previous study [18]. In brief, collected cultured media were centrifuged at 1000 ***g*** for 10 min at 4 °C to remove cellular debris. The supernatant fraction was centrifuged at 12 000 ***g*** for 30 min at 4 °C and further centrifuged at 110 000 ***g*** for 70 min at 4 °C. The pellet fraction was washed with PBS and then centrifuged at 110 000 ***g*** for 70 min at 4 °C again. The final precipitation was resuspended in PBS and stored at −80 °C until total RNA preparation. The protein concentrations of exosome fractions were quantified with a bicinchoninic acid (BCA) protein assay kit (Thermo Fisher Scientific K.K.).

### Observation of exosome fractions by electron microscopy

The exosome fractions were absorbed to carbon‐coated copper grids (400 mesh) and were stained with 2% phosphor‐tungstic acid solution (pH 7.0) for 10 s as a negative staining. The grids were observed with a transmission electron microscope (JEM‐1400Plus; JEOL Ltd., Tokyo, Japan) at an acceleration voltage of 100 kV. Digital images (3296 × 2472 pixels) were taken with a charge‐coupled device (CCD) camera (EM‐14830RUBY2; JEOL).

### Isolation of total RNA from the exosome fractions

Total RNA containing miRNA was extracted from the 3T3‐L1 cell‐derived exosome fractions using a mirVana miRNA isolation kit (Thermo Fisher Scientific). The quality of the RNA was evaluated with an Agilent Bioanalyzer 2100 with an RNA 6000 Pico Kit (Agilent Technologies, Santa Clara, CA, USA). First‐strand cDNA was synthesized with a miScript II RT kit (Qiagen, Tokyo, Japan).

### miRNA microarray analysis

For our microarray analysis of miRNA in exosome fractions, we conducted two independent experiments to collect cultured media (at D0, 4, 12, and 16 after changing to adipocyte DM‐1) as described above. Equal protein amounts of two exosomal miRNA samples from each sampling day were mixed, and cDNAs were synthesized. A single microarray analysis was conducted; that is, mixed cDNA samples were applied to a SurePrint G3 Mouse miRNA Microarray, release 19.0, 8x60K (Agilent) as described in our previous studies [18, 19]. Comparative analyses among samples were carried out. We selected miRNAs which altered expression levels with more than twofold or < 0.5‐fold at D4, D12, or D16 as compared to those at D0. Among them, we further selected miRNAs whose signal intensities were more than median values at any time points (12.3 at D0, 31.0 at D4, 24.4 at D12, and 17.7 at D16). Hierarchical cluster analysis was performed using complete linkage and Euclidean distance as measures of similarity. All data analysis and visualization of differentially expressed miRNA were conducted using r 3.1.2 (www.r‐project.org). Array data were deposited in the National Center for Biotechnology Information (NCBI) Gene Expression Omnibus (GEO) database (http://www.ncbi.nlm.nih.gov/geo). GEO Series accession number is GSE154875.

### Quantitative PCR assay of miRNAs

Quantitative PCR (qPCR) analyses were conducted using the QuantiTect SYBR Green PCR System (Qiagen) with a CFX96 thermal cycler (Bio‐Rad, Hercules, CA, USA) as described in our previous study [20] with slight modifications. The PCR program was 15 min at 95 °C, followed by 45 cycles of 15 s at 94 °C, 30 s at 55 °C, and 30 s at 70 °C. A miScript Primer Assay (Qiagen) was used to detect miR‐16‐5p, miR‐22‐3p, miR‐30d‐5p, miR‐34a‐5p, miR‐99a‐5p, miR‐125b‐5p, miR‐130a‐3p, miR‐214‐3p, and miR‐451a. The relative expression levels of miRNAs are shown as the ratios of ‘starting quantity’ to ‘protein content in exosome fraction’ and then normalized with values at D0. For qPCR of intracellular miRNA detection, RNU6‐2 (Qiagen) was used as an internal control RNA. The relative expression levels of target miRNAs to those of RNU6‐2 at D12 were normalized to the values at D0. Comparisons between groups were conducted by Student's *t*‐test. *P* values of < 0.05 were considered statistically significant.

### Gene ontology analysis

The target genes of miRNAs were predicted by TargetScanMouse (release 7.1; http://www.targetscan.org/mmu_71/). Predicted target genes with a cumulative weighted context++ score [21] of < 0.5 were selected for further gene ontology (GO) analysis. GO analysis of the predicted genes was performed by DAVID (ver. 6.8; https://david.ncifcrf.gov/home.jsp).

### Introduction of adipocyte‐derived exosomes into cultured skeletal muscle cells

Exosome fractions were labeled with PKH26 dye (Sigma‐Aldrich Japan K.K., Tokyo, Japan). Day 4 skeletal muscle cells following muscle differentiation were incubated with PKH26‐labeled exosome fraction for 16 h. As a negative control, the PKH26 dye labeling procedure was applied to PBS and the PBS was then added to culture medium. The cells were fixed with 4%‐paraformaldehyde phosphate buffer (Nacalai Tesque) for 20 min, rinsed three times with PBS, subsequently stained with Alexa Fluor 488 conjugated Wheat Germ Agglutinin (WGA; Thermo Fisher Scientific) for 20 min, and soaked with mounting media containing 4′,6‐diamidino‐2‐phenylindole (DAPI; Vector Laboratories, Inc., Burlingame, CA, USA). Samples were observed with an LSM 700 confocal laser scanning microscope (Carl Zeiss, Tokyo, Japan), equipped with a Plan‐Apochromat ×20 (NA 0.8) lens. Images were manipulated using imaging software (Carl Zeiss).

For exosome treatment experiments, the D0 and the D12+ adipocyte‐derived exosome fractions were used. Due to low concentrations of exosome fractions at late adipocyte differentiation stages, exosomes with equal amount of protein in each fraction (D12, D14, and D16) were mixed and designated as the D12+ exosome fraction. One day after muscle differentiation, cultured myoblasts were treated with the D0 exosome fraction (11 μg·mL^−1^), the D12+ exosome fraction (11 μg·mL^−1^), or PBS for 30 h and were harvested for gene expression analyses.

### Isolation of intracellular total RNA from cultured cells and qPCR assay

Total RNAs were isolated from differentiating 3T3‐L1 adipocytes and mouse myoblasts with an RNeasy kit (Qiagen). First‐strand cDNA was synthesized with a ReverTra Ace qPCR RT kit (Toyobo Co., Ltd., Osaka, Japan), and qPCR assay was performed as previously described [22]. The primer combinations used in the present study are shown in Table 1. qPCR was conducted using the QuantiTect SYBR Green PCR System (Qiagen) with a CFX96 Real‐Time PCR Detection System (Bio‐Rad, Tokyo, Japan). The qPCR protocol was the following: 15 min at 95 °C, followed by 45 cycles of 15 s at 94 °C, 30 s at 65 °C, and 30 s at 72 °C. All PCR products were verified by DNA sequencing (ABI 3730 DNA Analyzer; Life Technologies). The ratios of the expression levels of the target genes to ribosomal protein large subunit 7 (Rpl7) were calculated, and these ratios were then normalized with the expression levels of the PBS control groups. Rpl7 was used as an internal control gene since genes encoding for ribosomal proteins are the most stably expressed [23]. Multiple comparisons were conducted by analysis of variance (ANOVA), followed by *post hoc* test by least significant difference. *P* values of < 0.05 were considered statistically significant.

**Table 1:** PCR primer combinations.

Gene			Sequence (5′–3′)
*Adipoq*	NM_009605	Forward	ctgttcccaatgtacccattcgctttacta
		Reverse	ctctcaaactaagctgaaagtgtgtcgact
*Igf2*	NM_010514	Forward	gttgacacgcttcagtttgtctgttc
		Reverse	gacgtttggcctctctgaactcttt
*Myod1*	NM_010866	Forward	ctttgagacagcagacgacttctatgat
		Reverse	cagagcctgcagaccttcgatgtag
*Myog*	NM_031189	Forward	catgtaaggtgtgtaagaggaagtctgtgt
		Reverse	atggacgtaagggagtgcagattgt
*Myf5*	NM_008656	Forward	ctgagtacttctatgaaggctcctgtatcc
		Reverse	ctgtaatagttctccacctgttccctcag
*Myf6*	NM_008657	Forward	agaaaatgtgactcttcagccattagaagt
		Reverse	agaatttcttgcttgggtttgtagctgtag
*Myh3*	NM_001099635	Forward	gattcagaaactggagacacggatcagaga
		Reverse	tatacattctgcatatcttctgccctgcac
*Rpl7*	NM_001006345	Forward	catcagaattcgaggtatcaatggagtaag
		Reverse	ctccaccttctacaaagtgagttgtctttt

## Results

### Culturing of differentiating adipocytes

**Fig. 1:**
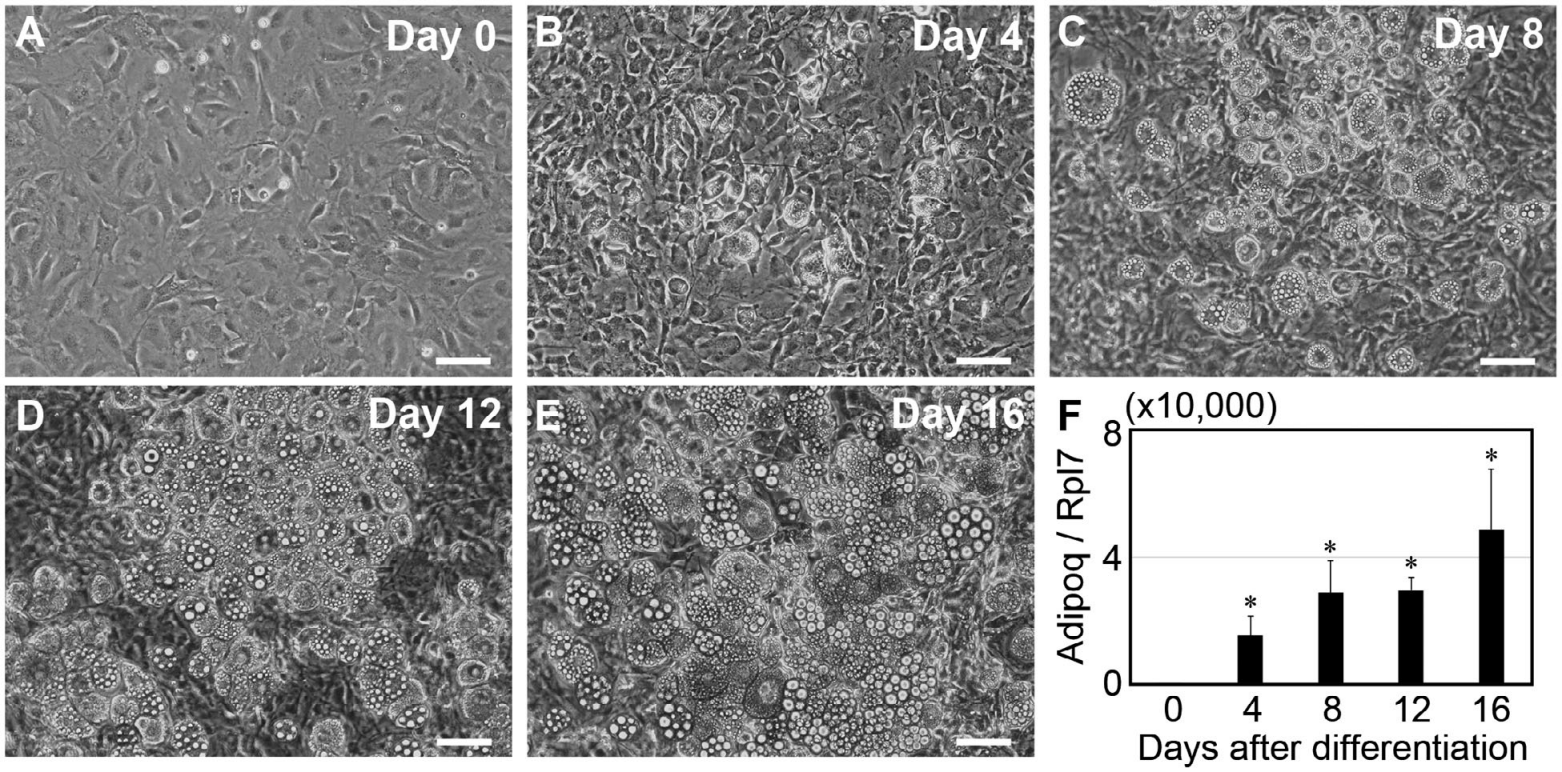
Culture results of mouse adipocyte 3T3‐L1 cells. (A–E) Representative photographs of differentiating adipocytes. Bars, 50 µm. (F) The relative expression levels of adiponectin (Adipoq), a typical adipocyte differentiation marker, were quantified by qPCR. Values represent the mean ± SE. *n* = 3. *P < 0.05 (vs D0), by Student's *t*‐test.

Mouse 3T3‐L1 cells were cultured to collect media containing exosomes. Intracellular small lipid droplets were observed in differentiating 3T3‐L1 cells from D4 onward. Gradually, these lipid droplets became larger at late culture stages (Fig. 1A–E). To evaluate adipocyte differentiation in cultured 3T3‐L1 cells, the mRNA expression levels of adiponectin (Adipoq), a typical adipocyte differentiation marker [24], were quantified by qPCR. The expression levels of Adipoq were upregulated as differentiation proceeded, indicating that cultured 3T3‐L1 cells were fully differentiated (Fig. 1F).

### Isolation of adipocyte‐derived exosomes

**Fig. 2:**
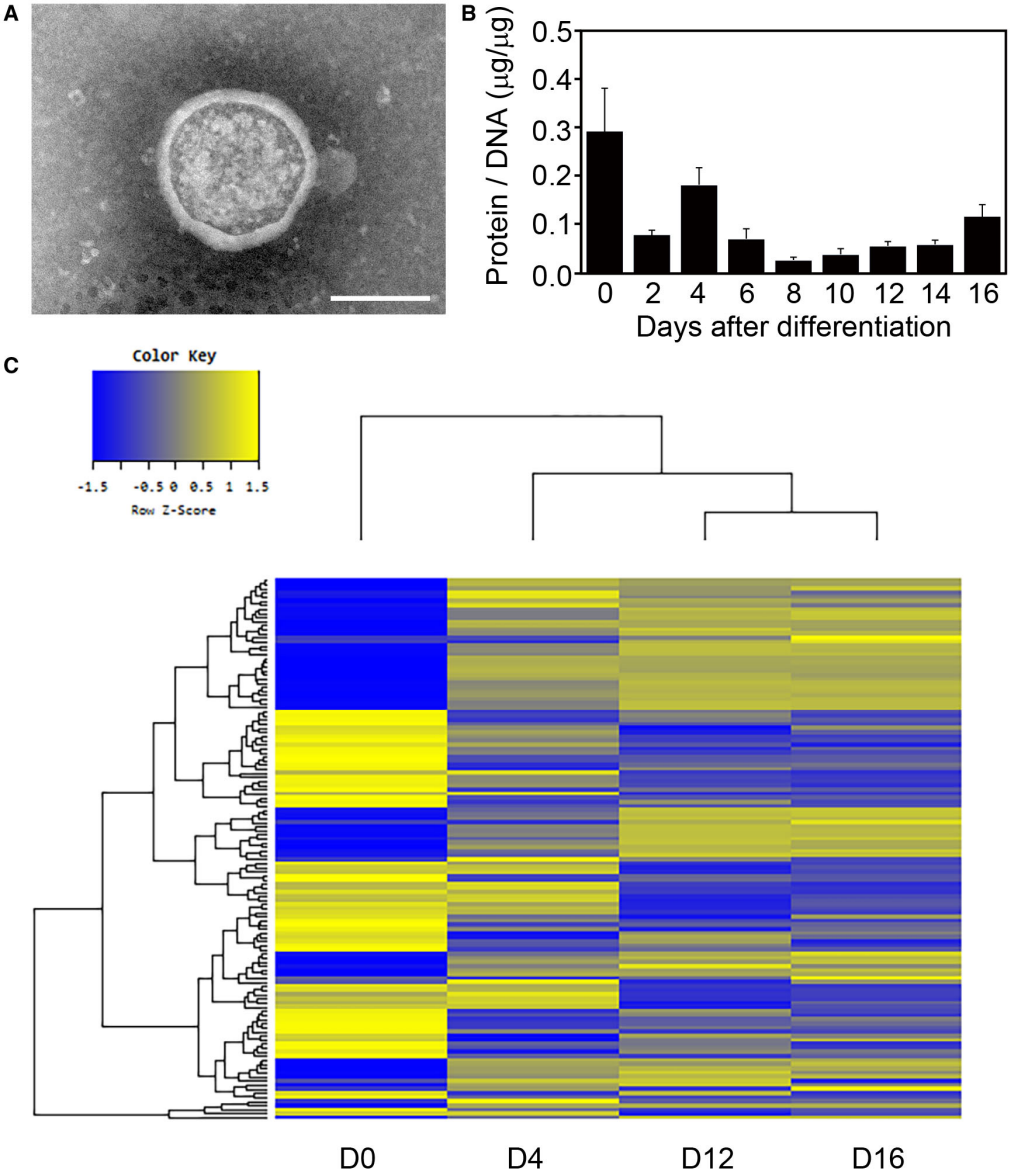
Characterization of adipocyte‐derived exosomes and exosomal miRNA. (A) Representative photograph of negatively stained adipocyte‐derived exosome fraction (D4) taken with transmission electron microscopy. Bar, 100 nm. (B) Amounts of adipocyte‐secreted exosome fraction during adipocyte differentiation. Protein concentrations in exosome fractions were normalized with the DNA contents of cultured cells. Values represent the mean ± SE, *n* = 3. (C) Expression changes of miRNA in adipocyte‐secreted exosomes during adipocyte differentiation (D0, D4, D12, and D16) were analyzed by hierarchical clustering.

Exosome fractions were isolated from 3T3‐L1 cultured media. To verify whether isolated fractions contained exosomes, negatively stained specimens were observed under electron microscopy. Typical secreted extracellular vesicles approximately 200 nm in diameter were observed in isolated exosome fraction (Fig. 2A), indicating that exosomes were present in our isolated exosome fraction. To quantify the released amounts of exosomes during adipocyte differentiation, protein contents in exosome fractions were measured (Fig. 2B). We found that distinct amounts of exosomes were secreted during adipocyte differentiation. The largest amount of exosome‐containing fraction was released at D0, with a second peak at D4. Although exosome fraction reached its lowest point at D8, the amounts of exosome fraction gradually increased during the process of further adipocyte differentiation.

### miRNA microarray analysis of adipocyte‐secreted exosome fractions

Microarray analysis was performed to gain an overview of the differentially expressed miRNAs in adipocyte‐secreted exosomes during adipocyte differentiation (D0, D4, D12, and D16). The numbers of detected miRNAs were 329, 426, 406, and 380 on D0, D4, D12, and D16, respectively. Hierarchical clustering analyses of miRNA expression profiles identified three groups of miRNAs in adipocyte‐secreted exosomes: the early (D0), middle (D4), and late differentiation stages (D12 and D16; Fig. 2C). Almost identical profiling patterns were observed on D12 and D16 (Pearson's correlation coefficient = 0.984).

### Expression patterns of adipocyte exosomal miRNAs

To assess the relative expression levels of miRNA in exosome fractions during adipocyte differentiation, the signal intensities of each miRNA were normalized with D0 signal intensities. In upregulated miRNAs, two main expression patterns were observed: UP‐I) gradual incremental increase during adipocyte differentiation, and UP‐II) reaching a plateau at D4 (Table 2). Downregulated miRNAs were categorized into three types: DOWN‐I) gradual decline during adipocyte differentiation, DOWN‐II) reaching maximum decrease at D4 and then maintaining their expression levels, and DOWN‐III) almost identical expression levels between D0 and D4 followed by a decrease at D12 (Table 3). Among the 406 miRNAs detected on D12, the number of upregulated miRNAs with more than twofold alteration (D12/D0) and the number of downregulated miRNAs with < 0.5‐fold alteration (D12/D0) were 41 and 35, respectively. These results indicate that maturing adipocytes dynamically change to release not only different amounts of exosomes but also different miRNA exosome contents.

**Table 2:** Upregulated miRNAs during 3T3‐L1 adipocyte differentiation. Upregulated miRNAs with more than twofold alteration (D12/D0) are listed.

Class	miRNA	D0/D0	D4/D0	D12/D0	D16/D0
UP‐I	miR‐451a	1.0	14.1	127.6	109.4
	miR‐99a‐5p	1.0	9.8	30.4	27.1
	miR‐322‐5p	1.0	6.4	15.9	13.5
	miR‐26b‐5p	1.0	6.2	13.3	13.3
	miR‐103‐3p	1.0	5.5	7.4	7.9
	miR‐193b‐3p	1.0	5.1	9.9	9.1
	miR‐26a‐5p	1.0	4.8	7.6	8.0
	miR‐25‐3p	1.0	4.2	7.2	6.5
	let‐7c‐5p	1.0	4.1	9.2	8.7
	miR‐107‐3p	1.0	4.1	5.9	6.3
	miR‐106b‐5p	1.0	4.0	8.2	7.9
	miR‐199b‐5p	1.0	3.5	6.2	5.9
	miR‐96‐5p	1.0	2.7	4.5	5.1
	miR‐16‐5p	1.0	3.8	4.9	4.5
	miR‐7070‐3p	1.0	3.4	3.5	4.4
	miR‐20a‐5p	1.0	2.9	4.9	4.8
	let‐7g‐5p	1.0	2.9	4.4	4.2
	miR‐19b‐3p	1.0	2.7	3.9	3.9
	miR‐15b‐5p	1.0	2.6	3.1	2.9
	let‐7d‐5p	1.0	2.6	4.0	3.3
	let‐7a‐5p	1.0	2.4	4.3	4.0
	miR‐92a‐3p	1.0	2.0	2.5	2.1
	miR‐23b‐3p	1.0	1.9	2.7	3.0
	miR‐130a‐3p	1.0	1.9	4.2	4.5
	let‐7f‐5p	1.0	1.8	2.9	3.0
	miR‐125b‐5p	1.0	1.7	3.3	3.4
	miR‐22‐3p	1.0	1.6	2.5	2.7
	let‐7i‐5p	1.0	1.5	2.6	2.5
UP‐II	miR‐30d‐5p	1.0	4.9	4.4	4.3
	miR‐669b‐3p	1.0	3.6	3.3	3.3
	miR‐199a‐3p	1.0	3.5	3.5	3.5
	miR‐342‐3p	1.0	3.3	3.2	3.4
	miR‐669l‐3p	1.0	3.2	3.1	3.2
	miR‐297a‐3p	1.0	3.2	3.1	3.1
	miR‐199a‐5p	1.0	3.0	3.0	2.7
	miR‐34a‐5p	1.0	2.9	2.7	2.5
	miR‐669d‐3p	1.0	2.7	2.5	2.5
	miR‐669i	1.0	2.7	2.6	2.5
	let‐7b‐5p	1.0	2.2	2.4	2.3
	miR‐7058‐3p	1.0	2.2	2.1	2.6
	miR‐125a‐5p	1.0	2.7	2.0	1.7

**Table 3:** Downregulated miRNAs during 3T3‐L1 adipocyte differentiation. Downregulated miRNAs with less than 0.5‐fold alteration (D12/D0) are listed.

Class	miRNA	D0/D0	D4/D0	D12/D0	D16/D0
Down‐I	miR‐7011‐5p	1.00	0.26	0.11	0.09
	miR‐1897‐5p	1.00	0.33	0.17	0.19
	miR‐8110	1.00	0.26	0.17	0.22
	miR‐1895	1.00	0.39	0.22	0.25
	miR‐5622‐3p	1.00	0.43	0.21	0.21
	miR‐7036a‐5p	1.00	0.94	0.37	0.50
	miR‐6385	1.00	0.86	0.28	0.38
	miR‐6360	1.00	0.84	0.45	0.48
	miR‐8101	1.00	0.76	0.45	0.59
	miR‐6898‐5p	1.00	0.71	0.17	0.21
	miR‐6368	1.00	0.71	0.42	0.72
	miR‐7686‐5p	1.00	0.67	0.23	0.23
	miR‐3098‐5p	1.00	0.66	0.47	0.61
	miR‐5126	1.00	0.65	0.34	0.37
	miR‐7118‐5p	1.00	0.63	0.15	0.14
	miR‐7005‐5p	1.00	0.62	0.21	0.32
	miR‐7082‐5p	1.00	0.47	0.28	0.41
	miR‐125a‐3p	1.00	0.94	0.10	0.20
Down‐II	miR‐466m‐5p	1.00	0.31	0.33	0.34
	miR‐669l‐5p	1.00	0.35	0.36	0.37
	miR‐669n	1.00	0.43	0.44	0.43
	miR‐574‐5p	1.00	0.43	0.48	0.46
	miR‐1187	1.00	0.45	0.47	0.47
	miR‐1906	1.00	0.26	0.36	0.32
	miR‐3470a	1.00	0.20	0.33	0.35
	miR‐669b‐5p	1.00	0.36	0.48	0.30
Down‐III	miR‐6769b‐5p	1.00	1.00	0.43	0.55
	miR‐3473g	1.00	1.07	0.43	0.52
	miR‐3473b	1.00	1.15	0.35	0.51
	miR‐29b‐3p	1.00	1.26	0.35	0.38
	miR‐5103	1.00	1.69	0.33	0.38
	miR‐7648‐3p	1.00	1.11	0.33	0.36
	miR‐188‐5p	1.00	1.30	0.30	0.29
	miR‐2137	1.00	1.09	0.21	0.29
	miR‐7047‐5p	1.00	1.29	0.31	0.26

### Validation of miRNA expression levels by qPCR

**Fig. 3:**
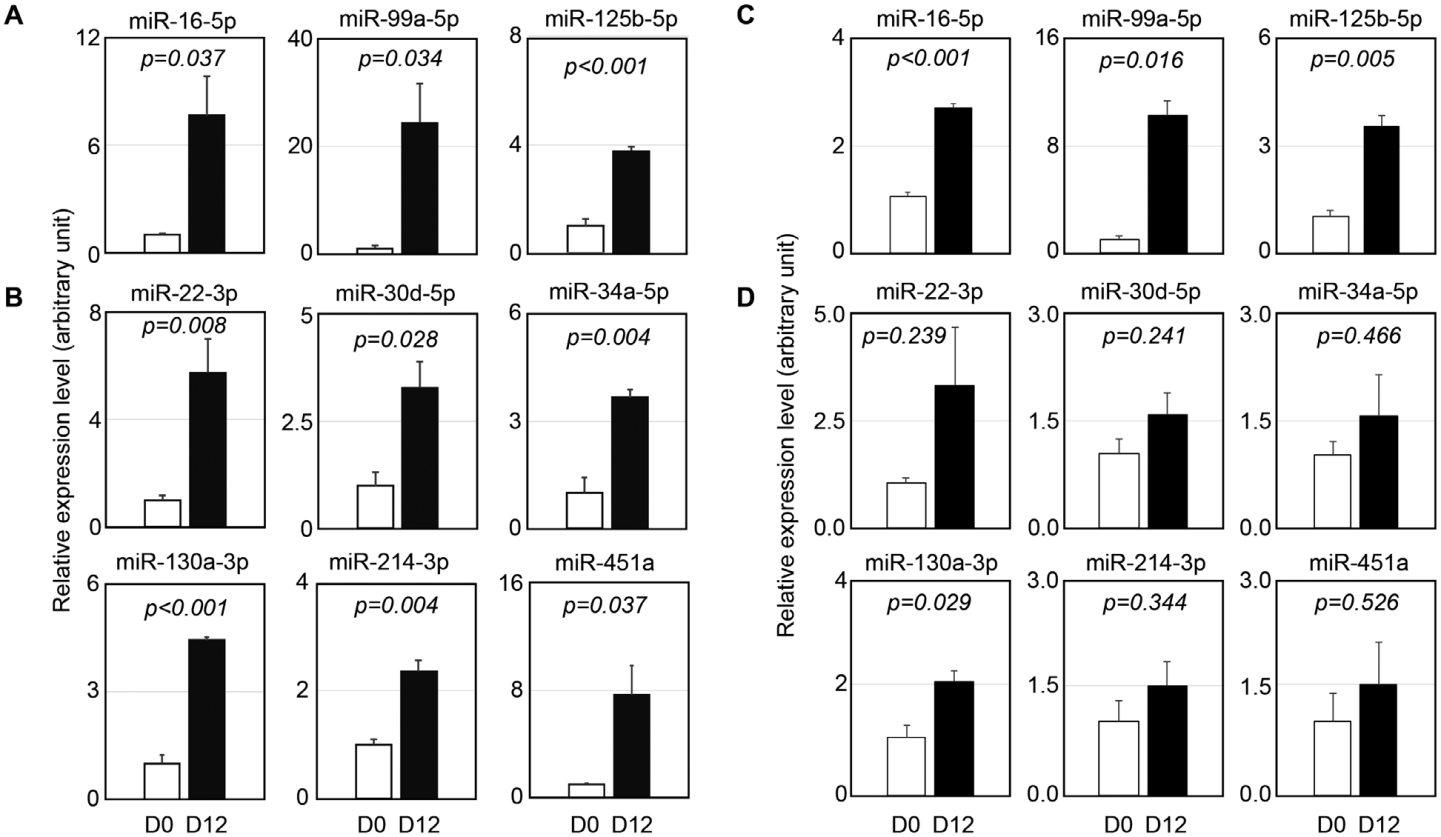
Validation of miRNAs in adipocyte‐derived exosomes and in adipocytes. (A, B) The relative expression levels of pro‐adipogenic miRNAs (A) and muscular dystrophy‐associated miRNAs (B) in exosome fractions were evaluated by quantitative PCR (qPCR). The relative expression levels of miRNAs at D12 were normalized to values at D0. Values represent the mean ± SE, *n* = 3. *P*‐values indicate the difference between D0 and D12 by Student's *t*‐test. (C, D) The expression levels of adipocyte intracellular miRNAs were assessed by qPCR. The ratios of expression levels of miRNAs regarding pro‐adipogenesis (C), and muscular dystrophy or muscle differentiation (D) to those of RNU6‐2 at D12 were normalized to values at D0. Values represent the mean ± SE, *n* = 3. *P*‐values indicate the difference between D0 and D12 by Student's *t*‐test.

To verify the expression levels of miRNA in adipocyte‐released exosome fractions, qPCR was carried out. qPCR revealed that the expression levels of known pro‐adipogenic miRNAs, including miR‐16‐5p, miR‐99a‐5p, and miR‐125b‐5p [25, 26, 27], were upregulated at D12 compared to D0 (Fig. 3A), suggesting that exosomal pro‐adipogenic miRNAs are delivered to recipient cells. We further validated miRNA expression levels found in the skeletal muscles of typical muscular dystrophy patients [28]. The expression levels of muscular dystrophy‐associated miRNAs such as miR‐34a‐5p, miR‐130a‐3p, and miR‐214‐3p were found to be significantly higher at D12 than at D0 (Fig. 3B). Other skeletal muscle differentiation‐associated miRNAs including miR‐22‐3p, miR‐30d‐5p, and miR‐451a were also found to have increased greatly at D12 (Fig. 3B).

To clarify whether the increased abundance of the miRNAs in the exosomes relates to changes in their expression in the adipocytes, the expression levels of intracellular miRNAs in D0 and D12 adipocytes were examined by qPCR experiments. Expression levels of pro‐adipogenic miRNAs such as miR‐16‐5p, miR‐99a‐5p, and miR‐125b‐5p were significantly higher in D12 adipocytes than in D0 adipocytes (Fig. 3C). In contrast to the pro‐adipogenic miRNAs, most of the intracellular miRNAs associated with muscular dystrophy and muscle differentiation were not significantly upregulated in D12 adipocytes (Fig. 3D). These results suggest that differentiating adipocytes may selectively release miRNAs as an exosome content, that is, in adipocytes, miRNAs which are associated with muscular dystrophy and muscle differentiation may be preferentially included into exosomes to be secreted.

### Gene ontology analyses of miRNA in adipocyte‐secreted exosomes

Our exosomal miRNA expression profile identified 41 miRNAs with more than twofold upregulation in the ratios of D12/D0. The target genes of these 41 miRNAs were predicted by Target Scan Mouse (release 7.1; http://www.targetscan.org/mmu_71/), and subsequently, 1956 genes were selected. Using 35 downregulated miRNAs, 2867 target genes were also predicted by Target Scan Mouse. GO analyses of these 1956 genes and 2867 genes were separately performed by DAVID (ver. 6.8; https://david.ncifcrf.gov/home.jsp). In both cases, GO terms associated with ‘transcriptional regulation’ accounted for 80% in the top five in predicted biological processes (Table 4 and 5). Intriguingly, predicted biological processes also included terms associated with skeletal muscle such as ‘skeletal muscle satellite cell maintenance’, and ‘muscle cell differentiation’, etc., although these terms were of lower rank in the lists of predicted biological processes (data not shown). These results suggest that adipocyte‐derived exosomes contain miRNAs that modulate the transcriptional regulation of skeletal muscle cell differentiation.

**Table 4:** GO terms extracted from the target genes of upregulated adipocyte exosomal miRNAs.

Term	Fold enrichment	Benjamini
GO:0000122~negative regulation of transcription from RNA polymerase II promoter	1.881	2.05E‐08
GO:0006355~regulation of transcription, DNA‐templated	1.435	4.55E‐08
GO:0006351~transcription, DNA‐templated	1.426	5.31E‐06
GO:0007264~small GTPase‐mediated signal transduction	2.418	4.94E‐06
GO:0045893~positive regulation of transcription, DNA‐templated	1.733	0.0002

**Table 5:** GO terms extracted from the target genes of downregulated adipocyte exosomal miRNAs.

Term	Fold enrichment	Benjamini
GO:0045944~positive regulation of transcription from RNA polymerase II promoter	1.518	4.35E‐06
GO:0006355~regulation of transcription, DNA‐templated	1.278	1.89E‐04
GO:0006351~transcription, DNA‐templated	1.307	1.98E‐04
GO:0016192~vesicle‐mediated transport	2.005	5.78E‐04
GO:0000122~negative regulation of transcription from RNA polymerase II promoter	1.496	0.0006

### Effect of adipocyte‐derived exosomes on skeletal muscle cells

**Fig. 4:**
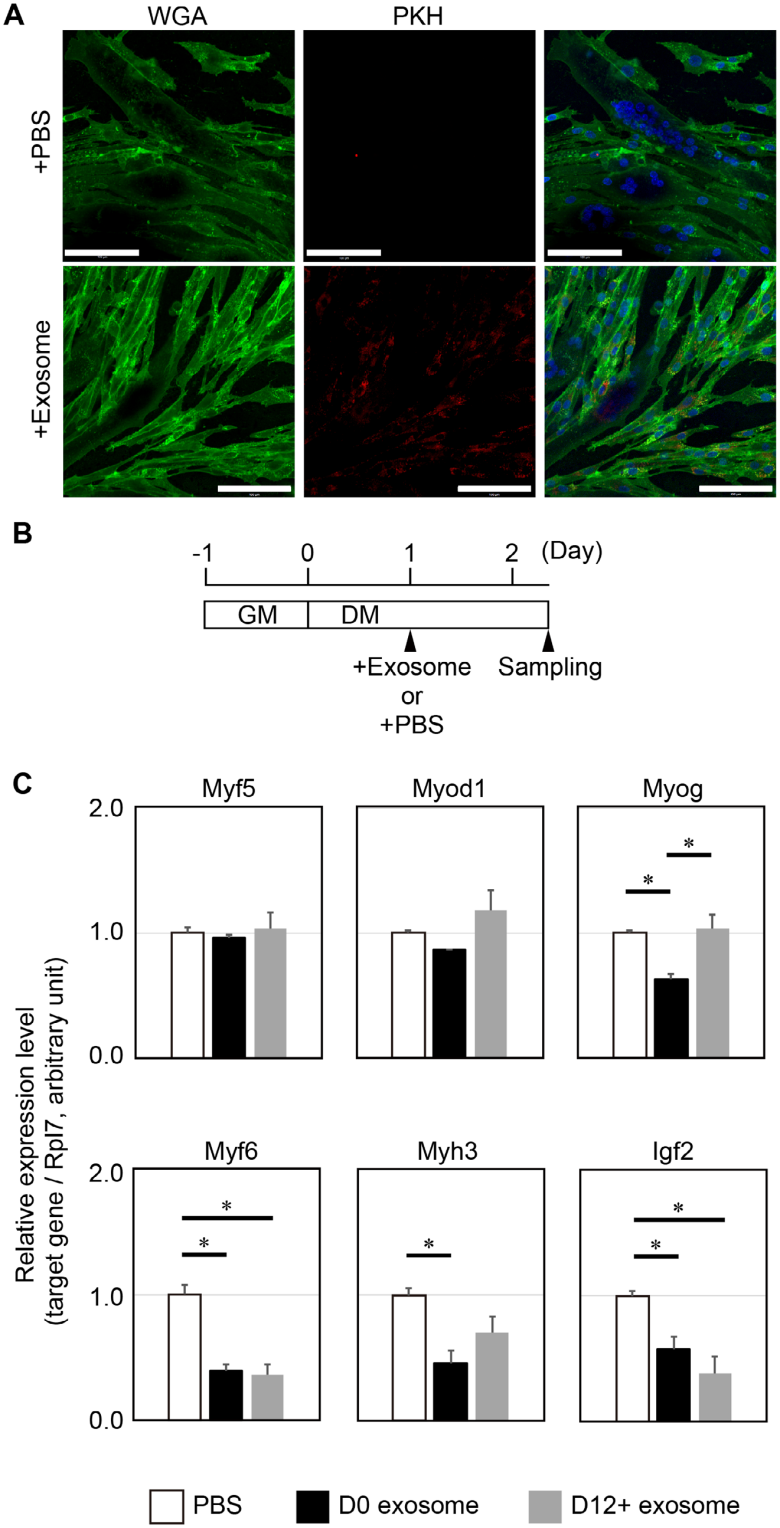
Effects of adipocyte‐derived exosome fractions on cultured muscle cells. (A) Adipocyte‐derived exosomes were incorporated into day 4 cultured muscle cells. Cells were incubated with PBS (+PBS) or adipocyte‐derived exosome fraction (+Exosome) for 16 h. Exosome fraction was labeled with PKH26 (red). Red indicated PKH26‐labeled exosomes which were incorporated into cells. The cells were stained with Alexa488‐conjugated WGA to visualize cell morphology (green). Nuclei were stained with Hoechst (blue). Bars, 100 µm. (B) A scheme of experimental design for exosome treatment was shown. Myoblasts were cultured in GM for 1 day and subsequently switched to DM. One day after shifting from GM to DM, myoblasts were treated with the D0 adipocyte‐derived exosome fraction, the D12+ adipocyte‐derived exosome fraction, or PBS as a control. D12+ exosome fraction contained the mixture of D12, D14, and D16 exosome fractions. Total RNAs were isolated from muscle cells following 30‐h treatment with exosome fractions or PBS. (C) Expression levels of genes regarding myogenic regulatory factors (Myf5, Myod1, Myog, and Myf6), Myh3, and Igf2 were quantified by qPCR. Differences in the expression ratios of the target gene to Rpl7 were compared among groups. Values represent the mean ± SE, *n* = 3. *P < 0.05, by ANOVA.

Given that exosomes function as cell‐to‐cell communication tools, we examined whether adipocyte‐derived exosomes were incorporated into cultured muscle cells. Isolated exosome fraction was labeled with PKH26 fluorescence dye, and dye‐labeled exosomes were then added to culture media. We observed PKH26‐positive mononucleated cells and myotubes following 16 h of incubation with exosomes (Fig. 4A). Interestingly, mononucleated cells tended to show more efficient uptake of exosomes.

To investigate the impact of adipocyte‐derived exosome treatment on the expression levels of myogenic regulatory factors (Myf5, Myod1, Myog, and Myf6), myoblasts were cultured for 30 h in the presence of one of the following fractions: the D0 adipocyte‐derived exosome fraction, the D12+ adipocyte exosome fraction which contained a mixture of D12, D14, and D16 adipocyte‐derived exosomes, or the PBS as a control (Fig. 4B). qPCR experiments revealed that the expression level of Myog was significantly downregulated in the D0 exosome‐treated group, although those of Myf5 and Myod1 were not changed among three groups (Fig. 4C). Importantly, the expression levels of Myf6 were significantly decreased in both the D0 and the D12+ exosome‐treated groups as compared with the PBS control group (Fig. 4C).

We further determined the expression levels of other muscle differentiation markers such as embryonic myosin heavy‐chain (Myh3) and insulin‐like growth factor 2 (Igf2). Myh3 is one of the early myofibrillar proteins during skeletal muscle development [29]. Igf2 expression is induced as early events in muscle differentiation [30] and reduction of Igf2 suppresses muscle differentiation [31]. The D0 exosome‐treated group significantly decreased Myh3 gene expression as compared to the PBS control group (Fig. 4C). Intriguingly, expression levels of the Igf2 gene were significantly decreased in myoblasts with the D0 and the D12+ exosome fraction treatments (Fig. 4C). Taken together, these results indicate that the D0 exosome fraction more effectively reduced the expression levels of genes associated with muscle differentiation than the D12 exosome fraction. Our findings also suggest that muscle differentiation was partially inhibited or retarded by the uptake of adipocyte‐derived exosomes.

## Discussion

As adipocyte miRNAs play a critical role in adipocyte differentiation and maturation [13, 14, 25, 32], most studies have focused on the functions of intracellular miRNAs in adipocytes. In the present study, we focused on extracellular miRNAs in exosomes secreted by differentiating adipocytes as a communication tool. Our adipocyte‐derived exosomal miRNA expression profiles showed that 3T3‐L1 adipocytes secreted not only different amounts of exosome fraction but also distinct types of exosomal miRNAs, including antimyogenic and pro‐adipogenic miRNAs, during adipocyte differentiation. These results imply that adipocyte‐derived exosomal miRNAs may worsen pathological conditions in recipient skeletal muscle cells *in vivo*.

### Partial suppression or delay of muscle differentiation by the uptake of adipocyte‐derived exosomes

Muscle differentiation is controlled by the precise hierarchical induction of Myf5 and Myod1, followed by Myog and Myf6. Myf5 and Myod1 function as muscle determination factors, whereas Myog and Myf6 are activated during myoblast differentiation to allow terminal differentiation [33, 34]. In our present study, molecular events associated with ‘transcriptional regulation’ were predicted by GO analysis based on adipocyte‐derived exosomal miRNA expression profile. In fact, we verified the downregulation of two myogenic regulation factor genes, Myog and Myf6, in myoblasts following adipocyte‐derived exosome treatment. This result suggests that adipocyte‐derived exosomes contained miRNAs to suppress muscle differentiation. It is interesting that the expression levels of Myog and Myf6 were specifically affected by adipocyte‐derived exosomes. Myog and Myf6 suppression might be explained by the timing when myoblasts were treated with exosome fractions. Myf5 and Myod1 are expressed in proliferating myoblasts [33]. Once muscle differentiation was induced, myoblasts stop proliferation and start to fuse each other to form myotubes, which is accompanied with expression of Myog and Myf6 [34]. Thus, muscle differentiation of day 1 myoblasts might be an appropriate timing to examine the effect of adipocyte‐derived exosomes on the expression of Myog and Myf6. Other than myogenic regulatory factors, the expression level of Myh3, also known as an embryonic Myh [29], was decreased in myoblasts treated with adipocyte‐derived exosome fraction. In other words, the D0 and the D12+ exosomes contained miRNAs to have effects on muscle differentiation process to modulate the gene expression of myofibrillar proteins as well as myogenic regulatory factors. Particularly, the D0 exosome fraction had an impact on Myog and Myh3 suppression in myoblasts as compared to the D12+ exosome fraction.

Igf2 is an embryonic regulator of myogenesis that promotes muscle differentiation [35]. Our results showed that myoblasts which were treated with adipocyte‐derived exosome fractions decreased the Igf2 expression. Intriguingly, Igf2 is one of the targets of miR‐125‐5p [31] that was contained in adipocyte‐derived exosomes (Table 2, Fig. 3). These results raise the possibility that muscle differentiation is retarded by the incorporation of adipocyte‐derived exosomal miR‐125b‐5p into myoblasts.

These results support the idea that adipocyte‐released exosomes function as an intercellular communication tool to carry miRNAs that suppress the expression of Myog and Myf6, although we have not identified which miRNAs are precisely involved in suppression of myogenic regulatory factors and Myh3. Downregulation of muscle differentiation‐associated genes may be attained with a combination of miRNAs in the adipocyte‐secreted exosomes as it is known that a single miRNA potentially has multiple target genes [36].

### Possible roles in the expansion of adipocytes in tissues through adipocyte‐derived exosomal miRNAs

One of the characteristic features of adipocytes is their expanding capacity in animal tissues. Ectopic intramuscular fat depositions are frequently observed within the perimysium of skeletal muscle tissues. Connective tissue surrounding adipocytes is disorganized and remodeled as adipocytes mature [37]. We found that adipocyte‐specific miRNAs such as miR‐16‐5p [27, 38], miR‐99a‐5p [25], and miR‐125b‐5p [26, 39] were included in adipocyte‐derived exosomes although these adipocyte‐specific miRNAs play a pro‐adipogenic role in adipocytes intracellularly [40]. Our results suggest that exosomal pro‐adipogenic miRNAs function not only intracellularly in adipocytes but also intercellularly in recipient adipocytes and their progenitors. In this context, adipocyte‐derived exosomes may facilitate adipocyte differentiation or the maturation of neighboring adipocytes and their progenitors in skeletal muscle tissues *in vivo*. As in the case of skeletal muscle tissues in muscular dystrophy patients and marbling cattle, it is believed that mature adipocytes secrete more exosomes with pro‐adipogenic miRNAs to enhance intramuscular fat deposition in skeletal muscle tissues (see below).

Eisenberg et al. detected consistently upregulated miRNAs in typical muscular dystrophy patients and believe these miRNAs to be implicated in the pathology of muscular dystrophy [28]. The present qPCR analysis confirmed that miRNAs, including miR‐34a‐5p, miR‐130a‐3p, and miR‐214‐3p, were upregulated in adipocyte‐derived exosomes, particularly at the matured adipocyte differentiation stage. In dystrophic muscles, muscle fibers atrophy and degenerate, while intramuscular connective tissues including adipocytes become dominant. In fact, fibrosis is deterioration by the upregulation of miR‐34a‐5p [41]. miR‐130a regulates the energy metabolism in adipose tissues [42], and miR‐214 is involved in muscle fate determination in zebrafish [43] and enhances adipocyte differentiation [44]. Therefore, these adipocyte‐derived exosomal miRNAs may worsen the pathological condition of skeletal muscle tissue in muscular dystrophy patients: adipocytes expand their area but the area of skeletal muscle cells decreases in the same skeletal muscle tissue. In other words, muscular dystrophy‐associated exosomal miRNAs secreted by adipocytes may be a key element in the worsening of muscle pathological conditions.

In skeletal muscle tissue with ectopic fat deposition, adipocytes predominantly accumulate in skeletal muscle tissue where the number and size of myofibers are reduced. This phenomenon might be explained in part by our experimental evidence that matured adipocytes proactively release exosomes to abrogate muscle differentiation, as described above. Furthermore, GO analysis using the target genes of adipocyte exosomal miRNA predicted biological processes regarding ‘satellite cell maintenance’, including muscle differentiation. Satellite cells are skeletal muscle tissue‐specific stem cells that regenerate myofibers [45]. However, muscle regenerating capacity is decreased in the muscles of muscular dystrophy patients [46, 47] and satellite cell density as well as myogenesis capability is reduced in the muscles of high marbling cattle [48]. These results suggest that adipocytes further expand their territory in skeletal muscle tissue to inactivate the maintenance of satellite cells using adipocyte‐released exosomal miRNAs.

Circulating exosomal miRNA in blood is linked to disease condition. For example, miR‐126 and miR‐223 are known markers of type 2 diabetes in human [49] and elevation of miR‐188 in sera of muscular dystrophy dog [50]. Furthermore, adipose tissue is a major source of circulating exosomal miRNAs [38]. In this context, miRNA markers for adipocyte invasion in skeletal muscle tissue should be present in the exosome fraction of sera from muscular dystrophy patients or marbling cattle. Our study contributes to the search for miRNA markers in exosomes that communicate between adipocytes and muscle cells.

## Author contributions

KOj, SM, and TN conceived and designed the research. KOj, SM, HW, and KOg performed the experiments; KOj, SM, and TN analyzed the data; and KOj, SM, MO, HW, KOg, and KT interpreted the results of experiments. KOj, SM, HW, and KOg prepared the figures, and SM and KOj drafted the manuscript. KOj, SM, MO, KOg and SM, and KT edited and revised the manuscript. KOj, SM, MO, HW, KOg, KT, and TN approved the final version of the manuscript.

## Conflict of interest

The authors declare no conflict of interest.

## Data Availability

The data that support the findings of this study are openly available in NCBI's Gene Expression Omnibus and are accessible through https://www.ncbi.nlm.nih.gov/geo/query/acc.cgi. GEO Series accession number is GSE154875.
